# Duration of Endocrine Treatment for DCIS impacts second events: Insights from a large cohort of cases at two academic medical centers

**DOI:** 10.21203/rs.3.rs-3403438/v1

**Published:** 2024-01-11

**Authors:** Thomas O’Keefe, Christina Yau, Emma Iaconetti, Eliza Jeong, Case Brabham, Paul Kim, Joseph McGuire, Ann Griffin, Anne Wallace, Laura Esserman, Olivier Harismendy, Gillian Hirst

**Affiliations:** Department of Surgery, University of California, San Diego; Department of Surgery, University of California, San Francisco, CA; Department of Surgery, University of California, San Francisco, CA; Moores Cancer Center, Division of Biomedical Informatics, UCSD School of Medicine University of California, San Diego, La Jolla, CA; Department of Surgery, University of California, San Francisco, CA; Department of Surgery, University of California, San Francisco, CA; UCSF Helen Diller Family Comprehensive Cancer Center; UCSF Helen Diller Family Comprehensive Cancer Center; Department of Surgery, University of California, San Diego; Department of Surgery, University of California, San Francisco, CA; Moores Cancer Center, Division of Biomedical Informatics, UCSD School of Medicine University of California, San Diego, La Jolla, CA; Department of Surgery, University of California, San Francisco, CA

**Keywords:** DCIS, treatment outcome, endocrine therapy, endocrine therapy duration

## Abstract

Ductal carcinoma in situ (DCIS) incidence has risen rapidly with the introduction of screening mammography, yet it is unclear who benefits from both the amount and type of adjuvant treatment (radiation therapy, (RT), endocrine therapy (ET)) versus what constitutes over-treatment. Our goal was to identify the effects of adjuvant RT, or ET+/− RT versus breast conservation surgery (BCS) alone in a large multi-center registry of retrospective DCIS cases (N = 1,916) with median follow up of 8.2 years. We show that patients with DCIS who took less than 2 years of adjuvant ET alone have a similar second event rate as BCS. However, patients who took more than 2 years of ET show a significantly reduced second event rate, similar to those who received either RT or combined ET+RT, which was independent of age, tumor size, grade, or period of diagnosis. This highlights the importance of ET duration for risk reduction.

## Introduction

Ductal carcinoma in situ (DCIS) has become a common diagnosis, particularly since the introduction of screening mammography, with an estimated 51,000 cases being diagnosed annually in the US in 2022 ^1^.The percentage of diagnosed breast neoplasms that are DCIS has dramatically increased since the pre-screening era (3% to 20%)^2^. However, it is not clear how much an earlier diagnosis has contributed to the reduction in breast cancer mortality when also considering improved treatments over the same time period, and what constitutes overdiagnosis^3,4^. DCIS presents as a biologically heterogeneous group of breast lesions which vary with respect to histopathologic features and outcomes^5^, yet the standard of care for all DCIS is surgery - either breast conserving surgery (BCS) or mastectomy due to the potential for the disease to progress to an invasive lesion^6^. This is followed typically (>80% cases) with adjuvant radiation therapy (RT). The evidence for adjuvant RT was based on the results of a series of randomized trials in the mid 1980’s to early 1990’s^7,8^ which showed that RT reduced the risk of a subsequent ipsilateral breast cancer by half. The addition of endocrine therapy (ET) to RT has been shown to have added benefit^9,10^ in terms of reduced risk of both ipsilateral and contralateral events but there is very limited information on the use of only adjuvant ET for DCIS^11,12,13^. Moreover, evidence from a number of small studies of unresected DCIS show between 14–53% of patients with DCIS lesions will not progress to invasive disease or death^14,15^. It has been demonstrated that endocrine therapy reduces the risk of invasive disease in the absence of any surgery in some DCIS cases^16^, lending support for active surveillance and ET in selected patients.

There is a need for improved algorithms for stratifying risk when considering treatment options for patients diagnosed with DCIS to reduce the potential harms of over-treatment. Additionally, there is a growing interest in the development of scoring systems using data from molecular, pathologic and imaging biomarkers to accomplish this. Two such scoring systems have been described to help guide treatment decisions^17,18^ but lack wide spread utilization.

One limiting factor is that few large databases exist for DCIS that contain adequate and consistent longitudinal data regarding clinicopathologic details and the outcomes of the patients studied. Another issue is that treatment specifics such as duration of ET, which is known to reduce recurrence (ipsilateral and contralateral) in invasive breast cancer^19^ has not been characterized in national registries or institutional studies of DCIS to date. It is quite likely that variability in the duration of ET had an impact on the conclusions drawn from historical trials^20^ which has influenced DCIS treatment practice to date. The Vermont DCIS registry^13^ described by Sprague and colleagues is one of the few studies which have examined the impact on second events of just adjuvant ET by itself following breast conservation surgery (BCS). For these reasons we wanted to conduct a deeper analysis of DCIS treatment and examine specifically the impact of ET duration on outcomes. For this purpose, we constructed a database for a large cohort of patients treated for DCIS at University of California San Francisco (UCSF) and San Diego (UCSD). This is tied into an ongoing tissue collection for downstream molecular and pathologic analysis. Cancer Registry data was confirmed by chart review and supplemented with durations of endocrine therapy for our final analysis cohort. We assessed time-varying risk of recurrence which included in situ and invasive ipsilateral and contralateral second breast cancer events by treatment type.

## Methods

### Patient Selection

Patients both diagnosed with and treated for index DCIS at UCSF and UCSD were identified through the respective cancer center registries. All patients identified were chart reviewed using the electronic medical record system with protocol approval from each center’s institutional review board. Inclusion criteria stipulated patients were at least 18 years of age at the time of diagnosis, underwent unilateral BCS or mastectomy for unilateral pure DCIS only, had no other malignancies, breast or otherwise, prior to their DCIS diagnosis and did not have a second event within 6 months of the initial DCIS surgery date. Additionally, criteria stipulated that patients undergoing BCS have some form of breast imaging at least 6 months after their DCIS surgery which was used to confirm presence of absence of second events, and patients undergoing mastectomy had a documented physical exam at least 6 months after their surgery to confirm presence or absence of second events. Last date of follow up was based on the most recent clinic note or imaging report. We did not include sex as an eligibility criterion, since male DCIS is treated similarly to female DCIS. However, DCIS in men is extremely rare representing approximately 9% of about 2700 male breast cancer cases annually based on SEER data^21^. 5 cases were included in our analysis cohort.

### Study Variables

We collected and chart reviewed: age at diagnosis of index DCIS, type of surgery performed, receipt of radiation therapy (RT), receipt of endocrine therapy (ET), duration of ET, lesion laterality, tumor grade, presence or absence of comedonecrosis, and estrogen receptor (ER) status. For each index DCIS we determined whether there was development of a second breast cancer event (SBCE) including either a DCIS recurrence or a progression to invasive disease, date of diagnosis and laterality of second event and most recent negative breast imaging for patients undergoing BCS or exam for patients undergoing mastectomy.

### Statistical Analysis

For patients who had a SBCE, the time between their initial DCIS diagnosis and their second event was calculated. For patients who did not have a SBCE, the time from their initial diagnosis to their last follow-up date was calculated. Follow up time is censored at 15 years. Each class of clinical variable (age, period of index diagnosis, size, grade, and ER status) were categorized into two or more groups for analysis; and associations with treatment groups (mastectomy vs. BCS, RT vs. No RT, and ET vs. No ET) were assessed using the chi-square test. Association between time to second event and clinical covariates as well as treatment type adjusting for clinical covariates, including age, period of diagnosis, lesion size and grade, and institution (UCSF vs. UCSD) was assessed using Cox proportional hazard models. Proportional hazard assumptions were tested using Schoenfeld residuals. Where appropriate, we used time dependent coefficients (for <7.5years and ≥7.5 years) to address violations in proportional hazard assumptions. In addition, we assessed the effects of RT and ET use on specific types of second events (ipsilateral invasive vs. ipsilateral in situ vs. contralateral) among patients who received BCS using competing risks models (with time dependent coefficients where appropriate). Statistical analysis was conducted using R package version 4.2.3.

## Results

### Treatment Cohorts

**Figure 1:**
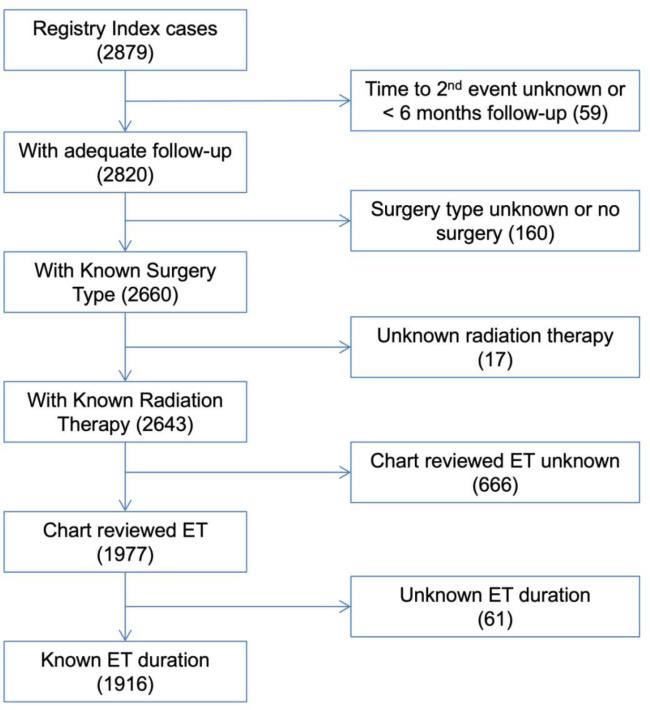
Study Consort

2,879 index DCIS cases were initially identified from the UCSF and UCSD cancer registries (Figure 1) covering the period from 1985 – 2017 through our selection criteria. Through subsequent extensive chart review, we excluded patients with missing data regarding surgical type, use of adjuvant RT or adjuvant ET, duration of ET, or with less than 6 months to a SBCE or less than 6 months of total follow up. Subsequently we identified 1,916 patients with pure index DCIS and treatment data in the electronic medical record. Median age at diagnosis was 55 years and median follow up time was 8.2 years. 539 (28%) patients in our cohort had a mastectomy with or without adjuvant ET. Among the patients who underwent BCS, 401 (21%) received BCS without adjuvant treatment (Table 1), 572 (30%) underwent BCS with RT, 152 (8%) underwent BCS with ET, and 252 (13%) underwent BCS with both ET and RT.

Treatment patterns changed over time with patients being more likely to receive adjuvant ET (p= 0.0018) or RT (p<0.0001) after 2001 than before (Table 1). Those who underwent mastectomy were more likely to be younger (median age 49) (p<0.0001), have DCIS of a larger size (p<0.0001), have high grade disease (p=0.0002), and have ER negative disease (p<0.0001) compared to those who underwent BCS. Among patients undergoing BCS, those who also had adjuvant RT (with or without adjuvant ET) were more likely to have higher grade (p<0.0001) and larger DCIS (p= 0.0009) than women in the groups that did not receive radiation.

Nearly three-quarters (N=296, 73%) of the patients who received BCS + ET (with or without RT, N=404), as well as 54% of patients (N=527) treated with BCS without adjuvant therapy or BCS with adjuvant RT had confirmed ER positive (ER+) disease. A large number of cases, (N=623, 33%) particularly those diagnosed in the earlier years of our study, did not have receptor data available (Table 1).

More than half of patients (70%) who received BCS with adjuvant ET alone or with RT continued their treatment for > 2 years, however a large majority (87%) of patients receiving mastectomy did not receive any form of ET. 257 of the 539 (76%) patients who had a mastectomy had recorded ER+ disease.

### Cumulative Incidence of Second Events by Treatment Type

1,916 patients received surgery (either BCS or mastectomy) with or without endocrine or radiation therapy, with median follow-up of 8.2 years. 194 patients (10%) experienced SBCE (Table 1) with the majority of events occurring in the first 6 years (Supplemental Table 1). Interestingly, none of the patient characteristics captured (period of diagnosis, lesion size, grade, presence of comedonecrosis and ER status), except age, was associated with risk of a second event. The risk of a second event was lower in the first 7.5 years and higher after 7.5 years in patients ≥50 when compared to those <50, reflecting differences in the timing of risk between younger and older patients (Supplemental Table 2).

**Figure 2:**
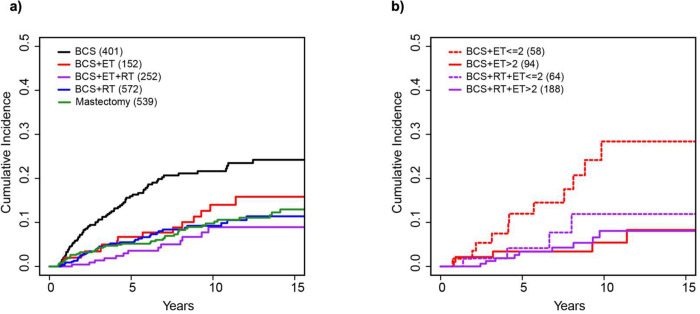
Cumulative Incidence of Any Second Event by Treatment Type. **a)** Cumulative incidence curves of second events by treatment types: BCS (black), BCS+ET (purple), BCS+RT (blue), Mastectomy (green). **b)** Cumulative Incidence curves for ET treated patients further stratified by duration of ET: BCS + ET ≤ 2 years (dotted red),BCS+ET > 2 years (solid red), BCS+RT+ET ≤ 2 years (dotted purple),BCS+RT+ET > 2 years (solid purple)

Cumulative incidence of SBCE was calculated, and when compared with BCS, patients in the other treatment groups had a significantly lower risk of developing any second event up to 15 years. (Figure 2A, Table 2). This risk reduction remains significant in a Cox multivariate model adjusting for age, size of lesion, grade, period of diagnosis and institution. (Table 2) Patients who were treated with only BCS had the highest estimated number of events by treatment type (16% at 5 years) which increased with time to 24% at 15 years (Table 2). In comparison, the estimated number of second events at 5 years was 5% in patients treated with adjuvant RT and 4% at 5 years with both adjuvant RT and ET. Similarly, patients treated with BCS and ET without RT had an estimated 7% risk of second events at 5 years. Although estimated risks increased over time for all treatment groups, there was still a reduced risk relative to BCS alone in both the BCS with RT (11%), BCS with RT and ET (9%), as well as BCS with ET (16%) treatment groups at 15 years.

Strikingly, when patients who received any ET were sub-divided into those who continued treatment for greater than 2 years and those who did not, significant risk reduction (relative to BCS) was only observed among those who received at least 2 years of treatment (Figure 2B, Table 3). This risk reduction was apparent in both patients who were treated with BCS with adjuvant ET (HR=0.15, p=0.008) as well as BCS with adjuvant RT and ET (HR=0.32, p=0.003).

The benefit of taking more than 2yrs of ET was also observed over time with an estimated 8% risk of second events at 15 years in both patients receiving BCS with ET (>2 years) and BCS with RT and ET (>2 years). In comparison, patients receiving ET for less than 2 years duration without RT had a 28% estimated risk of a SBCE at 15 years. Those receiving ET for less than 2 years but received adjuvant RT had a 12% risk, similar to the estimated risk seen in the BCS with RT treatment group (11%).

### Competing Risks for Type of Second Events in Patients receiving Breast Conservation Surgery

**Figure 3:**
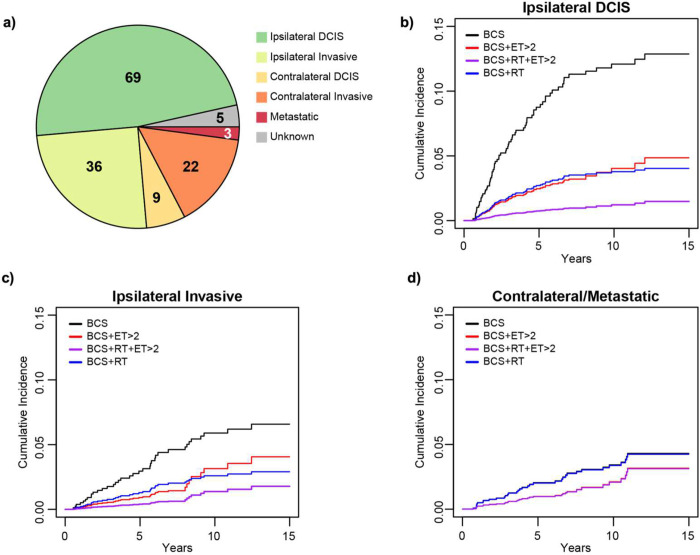
Type of second Event and Cumulative Incidence by Treatment Type. **a)** Distribution of type of second events among patients undergoing BCS. **(b-d)** Competing risk model predicted cumulative incidence curves of **(b)** Ipsilateral DCIS, (c) Ipsilateral Invasive and **(d)** Contralateral or Metastatic Second Events by Treatment Type. Patients who received ≤ 2 years of ET were grouped with those who did not receive ET: BCS (alone or with ET ≤ 2 years) (black), BCS+ET>2 (red), BCS+RT+ET>2(purple). BCS+RT (alone or with ET ≤ 2 years) (blue).

To understand the nature of second events following BCS we used a competing risks model with time varying coefficients to estimate risk of different event types as a function of ET and RT with censoring at 15 years. For this analysis we removed the 539 mastectomy cases and created a cohort of 1,377 patients treated with BCS with or without adjuvant treatment. Median follow up was 6.5 years. There were 144 second events with 3 being metastatic and 5 unknown events (Figure 3A). The majority of second events are ipsilateral (105/144, 73%) with 69 (66%) of them being DCIS. 31 second events (22%) were contralateral and a majority of these (71%) were invasive supporting the premise that DCIS is a marker of global risk.

Among all patients, the cumulative incidence of ipsilateral DCIS recurrence was 7% and the incidence of ipsilateral invasive and contralateral occurrence were both 4% at 15 years. In a multivariate competing risk model, (Table 4) RT significantly reduced the risk of both ipsilateral DCIS (HR= 0.29, p<0.0001) and ipsilateral invasive events (HR=0.42, p=0.01) as expected within the first 7.5 years compared to BCS without adjuvant therapy. This risk reduction was not observed between 7.5 and 15 years in our model. Without consideration of duration, any amount of ET also significantly reduced the risk of ipsilateral DCIS events within the first 7.5 years (HR= 0.44, p=0.02) (Table 4) and was associated with a non-significant risk reduction for ipsilateral invasive disease (HR=0.53, p=0.19) and contralateral DCIS (HR=0.43, p=0.12) in the first 7.5 years. In comparison, RT by itself was not associated with a risk reduction benefit for contralateral events (HR=0.97, p=0.93).

Figure 3B–D shows the predicted cumulative incidence curves for ipsilateral DCIS, ipsilateral invasive and contralateral or metastatic events respectively by treatment type, where ET is defined as taking greater than 2 years of ET. By stratifying patients undergoing BCS with ET into those receiving greater than 2 years of ET from those receiving less than 2 years, we see a significant further risk reduction (HR=0.27, p=0.0120) for ipsilateral DCIS second events and a possible further benefit for ipsilateral invasive events (HR=0.31, p=0.10), within the first 7.5 years only (Table 4). There was also an associated non-significant risk reduction in contralateral disease occurrence (HR=0.48, p=0.23).

## Discussion

In this study of index DCIS cases identified at two high volume academic medical centers, we describe treatment patterns over a 30-year period and conclude that adjuvant ET received for greater than 2 years can give the same reduction in risk for SBCE as adjuvant RT. The highlight of this study is that we conducted a detailed chart review of both receipt and duration of adjuvant endocrine therapy in over 1900 women, providing an important insight for practice considerations that has been lacking from other DCIS treatment outcome studies. Our data also suggests that ET by itself offers risk reduction for contralateral disease which is not observed with just RT. This supports the findings from Sprague et al ^13^ although our result was not statistically significant and is likely due to the small number of events. Limitations to this study included incomplete information for severable variables for inclusion in our model such as ER status, detailed histopathology such as comedonecrosis ^22^ which has been associated with risk of ipsilateral recurrence, and margin status especially for patients diagnosed before more comprehensive electronic health record data was available. However, margin status may be only relevant for BCS alone^23^. Another consideration is that early discontinuation of ET may have prognostic significance independent of the treatment effect, which cannot be adequately disentangled in a retrospective study. However it is well documented that extended ET duration has significant benefit in the treatment of ER+ invasive breast cancer^24^ and so our findings are not that unexpected .

Over the last twenty years, we have seen an increase in both breast cancer risk screening practice and diagnosis of DCIS as well as national treatment trends for more comprehensive adjuvant therapy in DCIS^25^. This is highlighted in our own institutional data where we show that the incidence of DCIS increased in later years accompanied with a rise in adjuvant RT with or without ET. SBCEs were only observed in 10% of women followed which is comparable with other institutional analyses^23,13^, however 22% of these events were contralateral and 2% were metastatic.

The critical question is how do we accurately stratify treatment for women for long term benefit? Natural history studies of DCIS without any treatment are rare^26,27,28^, but with the limited data we have from these small institutional studies as well as SEER suggest that untreated low-grade DCIS cases have a very low risk of invasive recurrence (~3–4% at 10yrs) whether they are surgically resected or not ^29,14,30^ as well as breast cancer specific survival at 10 years of 98% ^14^. Moreover, outcomes in SEER data show that the absolute risk of ipsilateral invasive cancer in all DCIS is 12.1% after 10 years without any definitive surgery at all, with risk that trends higher in patients with DCIS which is high grade, or ER-negative, or larger than 1cm ^30^ although not significantly.

Despite the evidence of a reservoir of lower risk disease ^31^, greater than 95% of DCIS is surgically removed in the US with approximately 25% of cases electing to have mastectomy according to SEER data up to 2016^32^. This is reflected in our own data where 28% of patients had mastectomy which tended to be clinically higher risk tumors with higher grade and presence of comedonecrosis. However, women with similarly higher risk tumors were also likely to be offered BCS with adjuvant RT, and we did not see any of our defined clinically high-risk features (high grade, comedonecrosis, age <50 or lesion size) associate with an increased risk of SBCE.

Our data confirms that adjuvant RT following BCS significantly reduces the risk of developing ipsilateral events, either in situ or invasive which is a direct effect of the nature of targeted radiotherapy to the involved breast. Similar to historical trials ^8^ and other studies^13, 33^ we show that the protective effect of RT on local risk is reduced after 5 years. It may be that the protective benefit of RT may be greatest for women with high-grade DCIS^34^ although several studies show no effect on overall survival ^11,7,35^. In our competing risks model group, we saw no additional benefit of RT for the risk of contralateral occurrence.

Unfortunately long term outcome data for DCIS patients treated with only adjuvant ET is mostly limited to non-specified sub-populations in a few trials^11 12 36^, and represents only 7–10% of treatment populations analyzed from registry studies ^13,37^. This is similar to the numbers in our cohort and highlights the challenge of getting enough cases for understanding the contribution of ET to risk reduction. Sprague et al.^13^ reported a long term benefit of adjuvant endocrine therapy over 15 years since DCIS diagnosis in their cohort of patients diagnosed in Vermont between 1994 and 2012 identified using a statewide registry and reported a benefit of ET on contralateral events up to 10 years. Their findings served in part as the inspiration for the present study, and taken together, our findings coupled with the results of this Vermont registry-based study suggest that rather than approaching ER+ DCIS from a therapeutic standpoint as a potential malignant precursor, it may be more beneficial to view it as a marker of increased risk for the development of eventual invasive disease. This would suggest that rather than focusing on local recurrence, as is done by RT, which reduces ipsilateral in situ and invasive recurrences within the first 5 years followed by a period of increased risk, we should adopt an approach of general prevention by means of the use of adjuvant endocrine therapy.

Importantly, we describe *duration* of ET on outcomes which is not reported in DCIS real world data such as that in SEER or routinely collected in registry studies since it requires additional detailed chart review. When we study the impact of adjuvant ET taken for 2 years or more, we see a similar reduction in risk of SBCEs comparable with RT only. We chose 2 years because it has been shown for early-stage invasive breast cancer that while 5 years of tamoxifen therapy in ER positive patients provides more protection than 1–2 years, the majority of benefit is received by two years^24,38^. Although we could not include ER status in our multivariate model because of missing data, we did note that women who were treated with ET were mostly ER+. As expected, we see the greatest risk reduction with the addition of ET to RT adjuvant therapy which is the current standard treatment recommendation for ER+ DCIS. However, importantly when we stratified receipt of ET to greater than 2 years, we saw the same reduced risk of 3% at 5 years which extended to 8% at 15 years whether adjuvant RT was received or not.

When we evaluated the effect of ET on the type and laterality of SBCE there was a significant reduction in risk of ipsilateral DCIS as well as a non-significant reduction in ipsilateral invasive disease with a hazard ratio of 0.53 which was further reduced among patients who received more than 2 years of ET. This was likely impacted by the number of events recorded and would be improved by larger datasets of adjuvant ET alone.

Risk of any second contralateral invasive cancer is estimated to be around 0.37% per year from a large analysis of SEER data of women with either DCIS or invasive cancer ^39^, although interestingly it is slightly higher for women with DCIS within the first 5 years. There is data to support that ET provide benefits beyond RT for contralateral events ^7 11, 12^ and our data showed a trend towards reduction of contralateral events with any ET, taken alone or with RT which was not seen with RT alone in the first 7.5 years.

There are several important factors that likely contribute to the benefits observed with adjuvant ET in our study which includes chart-reviewed duration of treatment and stratification of groups into those receiving at least two years of treatment or less since there were significant variations in durations of ET received in our cohort. We also showed that, where we could verify ER status, 70% of our ER+ patients did receive some form of ET, but very few ER- patients did which may contribute to the risk benefit we observed.

Given the estimated 3-fold increase in disease-specific mortality reported in SEER data^40^ for both treated and untreated DCIS patients, the risk of progression to invasive disease is an important aim of treatment. As with all clinical decision making, the risks and benefits of all therapies must be weighed for each patient, since both ET and RT are not without their adverse effects. Notably there is an increased risk of thromboembolic disease and endometrial cancer with conventional dosing of ET and long-term cardiac morbidity and poor cosmetic outcome with RT^41 42 43^. During chart review, many of our study patients were noted to have stopped their ET within weeks or months of initiation, most commonly due to intolerance of side effects.

DeCensi and Lazzeroni et al.^44,45^ have shown in recent results from the Tam01 study that low dose tamoxifen of 5mg taken daily for 3 years in ADH (atypical ductal hyperplasia), LCIS (Lobular carcinoma in situ) and DCIS patients reduced breast cancer second events by 50% with limited side-effects. Erikkson et al^46^ have also shown that a lower dose of tamoxifen has been associated with a dramatic reduction in symptoms in a breast cancer prevention setting for high risk women.

It is clear that women are confused by treatment options and their own personal risk ^47^, which is exemplified by the poor recruitment to active monitoring trials LORIS and LORD which offered randomization to surveillance and annual mammogram for low-risk DCIS ^48^ and are now being re-designed. Efforts in biomarker development and testing for additional markers such as HER2 in DCIS for risk stratification,^49 50 51^ combined with evidence for the use of more tolerable endocrine therapies for both invasive breast cancer and DCIS ^52,53^ should refine our treatment approaches. Patients, surgeons, and medical oncologists think that there is little evidence for supporting the use of ET at the time of a diagnosis of DCIS. In fact, women with ER+ DCIS may be the very patients with elevated risk for an invasive cancer event where endocrine risk reducing therapy is effective ^54^. The results of our real-world outcomes data analysis support the use of ET for risk reduction which provides equivalent long term risk benefit as RT when well tolerated. Additional benefit is likely for contralateral risk reduction, and it is our view that ET should be considered in both the adjuvant and neoadjuvant context for treatment of DCIS.

## Supplementary Material

Supplement 1

## Figures and Tables

**Table 1:** Patient Characteristics

	BCS (N=401)	BCS+RT (N=572)	BCS+ET (N=152)	BCS+ET+RT (N=252)	Mastectomy +/− ET (N=539)	Overall (N=1916)
**Age**						
Median (Range)	59 (30–95)	59 (34–84)	55.5 (19–83)	54.5 (28–78)	49 (24–81)	55 (19 – 95)
<50	102 (25.4%)	123 (21.5%)	48 (31.6%)	79 (31.3%)	285 (52.9%)	637 (33.2%)
>=50	299 (74.6%)	449 (78.5%)	104 (68.4%)	173 (68.7%)	254 (47.1%)	1279 (66.8%)
**Period of Index Diagnosis**						
1985–1993	17 (4.2%)	6 (1%)	1 (0.7%)	1 (0.4%)	28 (5.2%)	53 (2.8%)
1994–2001	92 (22.9%)	56 (9.8%)	16 (10.5%)	18 (7.1%)	97 (18%)	279 (14.6%)
2002–2009	136 (33.9%)	204 (35.7%)	68 (44.7%)	106 (42.1%)	190 (35.3%)	704 (36.7%)
2010–2017	156 (38.9%)	306 (53.5%)	67 (44.1%)	127 (50.4%)	224 (41.6%)	880 (45.9%)
**Size**						
<= 2cm	228 (80%)	332 (73.6%)	86 (84.3%)	130 (67.4%)	187 (47.7%)	963 (67.7%)
>2cm	57 (20%)	119 (26.4%)	16 (15.7%)	63 (32.6%)	205 (52.3%)	460 (32.3%)
Unknown	116	121	50	59	147	493
**Grade**						
Low	31 (9.3%)	29 (5.8%)	18 (13.6%)	12 (5.1%)	23 (4.9%)	113 (6.8%)
Intermediate	162 (48.8%)	187 (37.6%)	71 (53.8%)	108 (46%)	166 (35.7%)	694 (41.8%)
High	139 (41.9%)	282 (56.6%)	43 (32.6%)	115 (48.9%)	276 (59.4%)	855 (51.4%)
Unknown	69	74	20	17	74	254
**Comedonecrosis**						
No	127(62.3%)	181(50.1%)	44(65.7%)	67(42.4%)	115(36.9%)	534 (48.5%)
Yes	77(37.7%)	180(49.9%)	23(34.3%)	91(57.6%)	197(63.1%)	568 (51.5%)
Unknown	197	211	85	94	227	814
**ER Status**						
Positive	192 (86.1%)	335 (79.2%)	100 (98%)	196 (95.6%)	257 (75.6%)	1080 (83.5%)
Negative	31 (13.9%)	88 (20.8%)	2 (2%)	9 (4.4%)	83 (24.4%)	213 (16.5%)
Unknown	178	149	50	47	199	623
**Endocrine Therapy Duration**						
<=2	0	0	58 (38.2%)	64 (25.4%)	33 (6.1%)	155 (8.1%)
>2	0	0	94 (61.8%)	188 (74.6%)	36 (6.7%)	318 (16.6%)
No ET	401 (100%)	572 (100%)	0	0	470 (87.2%)	1443 (75.3%)
**Institution**						
UCSD	160 (40%)	363 (63%)	56 (37%)	170 (67%)	247 (46%)	996 (52%)
UCSF	241 (60%)	209 (37%)	96 (63%)	82 (33%)	292 (54%)	920 (48%)
**Follow-up**						
Time to last follow-up, Median (Range), yrs	9.2 (0.6–32.8)	6.7 (0.5–29.6)	9.4 (0.6–24.6)	7.6 (0.5–28.6)	9.0 (0.5–31.1)	8.2 (0.5–32.8)
Number of second events	75	41	16	15	47	194

**Table 2:** Association between Second Events and Treatment Type

	Univariate			Multivariate*			Kaplan Meier Estimates of (any) Second Event Rate		
	N	Hazard Ratio (95% CI)	Wald test p	N	Hazard Ratio (95% CI)	Wald test p	5yr	10yr	15yr
**Treatment type (5-level factor)**									
BCS	401	REF		257	REF		16% (12%-20%)	22% (17%-26%)	24% ( 19%-29%)
BCS+RT	572	0.40 (0.27–0.59)	<0.0001	407	0.34 (0.2–0.56)	<0.0001	5% (3%-7%)	9% (6%-12%)	11% (8%-15%)
BCS+(any)ET	152	0.53 (0.31–0.91)	0.0208	96	0.37 (0.18–0.78)	0.0094	7% (2%-11%)	14% (7%-21%)	16% (8%-23%)
BCS+(any)ET+RT	252	0.27 (0.15–0.5)	<0.0001	186	0.34 (0.17–0.65)	0.0014	4% ( 1 %-6%)	9% (4%-14%)	9% (4%-14%)
Mastectomy	539	0.41 (0.29–0.6)	<0.0001	357	0.32 (0.2–0.53)	<0.0001	5% (3%-7%)	10% (7%-13%)	13% (9%-17%)

* Multivariate model adjusting for age, period of diagnosis, tumor size, grade and institution, with time dependent coefficients for age and size

**Table 3:** Association between Second Events and Treatment Type stratified by Endocrine Therapy Duration (<2yr vs. >2yrs)

	Univariate			Multivariate*			Kaplan Meier Estimates of (any) Second Event Rate		
	N	Hazard Ratio (95% CI)	Wald test P	N	Hazard Ratio (95% CI)	Wald test p	5yr	10yr	15yr
**Treatment type (7-level factor)**									
BCS	401	REF		257	REF		16% (12%-20%)	22% (17%-26%)	24% (19%-29%)
BCS+RT	572	0.40 (0.27–0.59)	<0.0001	407	0.33 (0.2–0.56)	<0.0001	5% (3%-7%)	9% (6%-12%)	11% (8%-15%)
BCS+ET(<=2)	58	1.00 (0.53–1.88)	0.9961	35	0.75 (0.32–1.75)	0.5039	12% (2%-21%)	28% (11 %-42%)	28% (11 %-42%)
BCS+ET(>2)	94	0.26 (0.1–0.64)	0.0035	61	0.15 (0.04–0.6)	0.0078	3% (0%-7%)	5% (0%-11%)	8% (0%-16%)
BCS+RT+ET(<=2)	64	0.39 (0.14–1.06)	0.0642	45	0.39 (0.12–1.25)	0.1139	4% (0%-10%)	12% (0%-23%)	12% (0%-23%)
BCS+RT+ET(>2)	188	0.24 (0.12–0.49)	0.0001	141	0.32 (0.15–0.68)	0.0031	3% (0%-6%)	8% (3%-13%)	8% (3%-13%)
Mastectomy	539	0.41 (0.29–0.6)	<0.0001	357	0.32 (0.2–0.53)	<0.0001	5% (3%-7%)	10% (7%-13%)	13% (9%-17%)

* Multivariate model adjusting for age, period of diagnosis, tumor size, grade and institution, with time dependent coefficients for age and size

**Table 4:** Competing Risk Analysis of Type of Second Event by Treatment Type

		Hazard Ratio (CI)	p			Hazard Ratio (CI)	p
**Ipsilateral Invasive**							
RT		0.42 (0.21–0.83)	0.01	RT		0.43 (0.22–0.85)	0.02
(any)ET*	0 – 7.5 yr	0.53 (0.2–1.38)	0.19	ET>2yr*	0 – 7.5 yr	0.31 (0.07–1.27)	0.10
	7.5 – 15 yr	1.46 (0.35–6.01)	0.60		7.5 – 15 yr	1.30 (0.26–6.35)	0.75
**Ipsilateral DCIS**							
RT		0.29 (0.17–0.48)	<0.0001	RT		0.30 (0.18–0.5)	<0.0001
(any)ET*	0 – 7.5 yr	0.44 (0.23–0.87)	0.02	ET>2yr*	0 – 7.5 yr	0.27 (0.1–0.75)	0.01
	7.5 – 15 yr	9.73 (1.13–83.77)	0.04		7.5 – 15 yr	0.97 (0.11–8.65)	0.98
**Contralateral DCIS**							
RT		0.97 (0.49–1.91)	0.93	RT		0.99 (0.5–1.93)	0.97
(any)ET*	0 – 7.5 yr	0.43 (0.15–1.23)	0.12	ET>2yr*	0 – 7.5 yr	0.48 (0.14–1.61)	0.23
	7.5 – 15 yr	1.39 (0.34–5.78)	0.65		7.5 – 15 yr	1.18 (0.25–5.69)	0.83

* ET effect modeled using time dependent coefficients

## Data Availability

All of the data for this study are available to investigators upon reasonable request.
