# Supplementary material for: Duration of Endocrine Treatment for DCIS impacts second events: Insights from a large cohort of cases at two academic medical centers
Source: Res Sq. 2024 Jan 11:rs.3.rs-3403438. Preprint. [Version 1] doi: 10.21203/rs.3.rs-3403438/v1 (PMC10802747; doi:10.21203/rs.3.rs-3403438/v1)
Supplement: Supplement 1 [file NIHPPrs3403438v1-supplement-1.pdf]

Supplemental Table 1: Event Counts

Year	BCS	BCS+RT	BCS+ET	BCS+ET+RT	Mastectomy	Total
**0-1**	**10**	**5**	**3**	**0**	**9**	**27**
Unknown	0	1	0	0	0	1
Ipsilateral Invasive	2	0	1	0	4	7
Ipsilateral DCIS	6	1	1	0	0	8
Contralateral/Metastatic	2	3	1	0	5	11
**1-2**	**19**	**8**	**1**	**1**	**8**	**37**
Unknown	0	0	0	0	0	0
Ipsilateral Invasive	5	2	0	0	4	11
Ipsilateral DCIS	13	5	1	0	0	19
Contralateral/Metastatic	1	1	0	1	4	7
**2-3**	**11**	**9**	**1**	**2**	**5**	**28**
Unknown	3	1	0	0	0	4
Ipsilateral Invasive	2	0	1	0	0	3
Ipsilateral DCIS	6	5	0	1	1	13
Contralateral/Metastatic	0	3	0	1	4	8
**3-4**	**8**	**4**	**2**	**1**	**2**	**17**
Unknown	0	0	0	0	0	0
Ipsilateral Invasive	2	1	0	1	1	5
Ipsilateral DCIS	3	1	2	0	0	6
Contralateral/Metastatic	3	2	0	0	1	6
**4-5**	**8**	**1**	**2**	**3**	**2**	**16**
Unknown	0	0	0	0	0	0
Ipsilateral Invasive	1	1	0	1	0	3
Ipsilateral DCIS	4	0	2	2	1	9
Contralateral/Metastatic	3	0	0	0	1	4
**5-6**	**6**	**4**	**1**	**0**	**0**	**11**
Unknown	0	0	0	0	0	0
Ipsilateral Invasive	2	2	1	0	0	5
Ipsilateral DCIS	4	2	0	0	0	6
Contralateral/Metastatic	0	0	0	0	0	0
**6-7**	**6**	**5**	**0**	**2**	**4**	**17**
Unknown	0	0	0	0	0	0
Ipsilateral Invasive	2	1	0	0	1	4
Ipsilateral DCIS	3	1	0	1	0	5
Contralateral/Metastatic	1	3	0	1	3	8
**7-8**	**1**	**0**	**1**	**0**	**6**	**8**
Unknown	0	0	0	0	0	0
Ipsilateral Invasive	0	0	0	0	0	0
Ipsilateral DCIS	0	0	0	0	0	0
Contralateral/Metastatic	1	0	1	0	6	8
**8-9**	**0**	**2**	**2**	**2**	**2**	**8**
Unknown	0	0	0	0	0	0

Ipsilateral Invasive	0	2	0	2	0	4
Ipsilateral DCIS	0	0	2	0	0	2
Contralateral/Metastatic	0	0	0	0	2	2
**9-10**	**1**	**0**	**2**	**2**	**2**	**7**
Unknown	0	0	0	0	1	1
Ipsilateral Invasive	1	0	1	0	0	2
Ipsilateral DCIS	0	0	1	0	0	1
Contralateral/Metastatic	0	0	0	2	1	3
**10-11**	**3**	**2**	**0**	**0**	**2**	**7**
Unknown	0	0	0	0	1	1
Ipsilateral Invasive	1	0	0	0	0	1
Ipsilateral DCIS	0	0	0	0	0	0
Contralateral/Metastatic	2	2	0	0	1	5
**11-12**	**0**	**0**	**1**	**0**	**1**	**2**
Unknown	0	0	0	0	0	0
Ipsilateral Invasive	0	0	0	0	0	0
Ipsilateral DCIS	0	0	1	0	0	1
Contralateral/Metastatic	0	0	0	0	1	1
**12-13**	**1**	**1**	**0**	**0**	**0**	**2**
Unknown	0	0	0	0	0	0
Ipsilateral Invasive	1	0	0	0	0	1
Ipsilateral DCIS	0	1	0	0	0	1
Contralateral/Metastatic	0	0	0	0	0	0
**13-14**	**0**	**0**	**0**	**0**	**2**	**2**
Unknown	0	0	0	0	0	0
Ipsilateral Invasive	0	0	0	0	0	0
Ipsilateral DCIS	0	0	0	0	0	0
Contralateral/Metastatic	0	0	0	0	2	2
**14-15**	**0**	**0**	**0**	**0**	**1**	**1**
Unknown	0	0	0	0	0	0
Ipsilateral Invasive	0	0	0	0	0	0
Ipsilateral DCIS	0	0	0	0	0	0
Contralateral/Metastatic	0	0	0	0	1	1
**>15***	**1**	**0**	**0**	**2**	**1**	**4**
Unknown	0	0	0	0	0	0
Ipsilateral Invasive	0	0	0	1	0	1
Ipsilateral DCIS	0	0	0	0	0	0
Contralateral/Metastatic	1	0	0	1	1	3
**Total**	**75**	**41**	**16**	**15**	**47**	**194**
Unknown	3	2	0	0	2	7
Ipsilateral Invasive	19	9	4	5	10	47
Ipsilateral DCIS	39	16	10	4	2	71
Contralateral/Metastatic	14	14	2	6	33	69

*** Second events occurring after 15 years are censored from analysi**

Supplemental Table 2: Association between Second Events and Clinical Covariates

	N	Hazard Ratio		LCI	UCI	Wald test p
**Age**						
Age < 50	637		REF			
Age >= 50	1279	0-7.5 yr	0.62	0.45	0.85	0.003
		7.5-15 yr	2.45	1.07	5.62	0.034
**Period of Diagnosis**						
1985-1993	53		1.03	0.46	2.3	0.948
1994-2001	279		1.45	0.95	2.22	0.084
2002-2009	704		1.12	0.77	1.63	0.553
2010-2017	880		REF			
**Size**						
<= 2cm	460		REF			
>2cm	963	0-7.5 yr	1.2	0.81	1.77	0.367
		7.5-15 yr	0.7	0.26	1.9	0.487
**Grade**						
Grade I/II	807		REF			
Grade III	855		1.18	0.86	1.62	0.3
**Comedonecrosis**						
Absent	534		REF			
Present	568		1.29	0.84	1.96	0.245
**ER status**						
ER Positive	1080		REF			
ER Negative	213		1.08	0.65	1.81	0.76

LCI-Lower Confidence Interval, UCI-Upper Confidence Interval
